# Possible Association Between DHEA and PKCε in Hepatic Encephalopathy Amelioration: A Pilot Study

**DOI:** 10.3389/fvets.2021.695375

**Published:** 2021-09-28

**Authors:** Alessandro Di Cerbo, Luca Roncati, Carlotta Marini, Gianluca Carnevale, Manuela Zavatti, Rossella Avallone, Lorenzo Corsi

**Affiliations:** ^1^School of Biosciences and Veterinary Medicine, University of Camerino, Matelica, Italy; ^2^Institute of Pathology, University of Modena and Reggio Emilia, Modena, Italy; ^3^Surgical, Medical and Dental Department of Morphological Sciences Related to Transplant, Oncology and Regenerative Medicine, University of Modena and Reggio Emilia, Modena, Italy; ^4^Department of Biomedical, Metabolic and Neural Sciences, University of Modena and Reggio Emilia, Modena, Italy; ^5^Department of Life Sciences, University of Modena and Reggio Emilia, Modena, Italy; ^6^National Institute of Biostructure and Biosystems, Rome, Italy

**Keywords:** hepatic encephalopathy, hyperammonemia, protein kinase C (PKC), DHEA, animal model

## Abstract

**Objective:** Hepatic encephalopathy (HE) is a neuropsychiatric syndrome caused by liver failure and by an impaired neurotransmission and neurological function caused by hyperammonemia (HA). HE, in turn, decreases the phosphorylation of protein kinase C epsilon (PKCε), contributing to the impairment of neuronal functions. Dehydroepiandrosterone (DHEA) exerts a neuroprotective effect by increasing the GABAergic tone through GABA_A_ receptor stimulation. Therefore, we investigated the protective effect of DHEA in an animal model of HE, and the possible modulation of PKCε expression in different brain area.

**Methods:** Fulminant hepatic failure was induced in 18 male, Sprague–Dawley rats by i.p. administration of 3 g/kg D-galactosamine, and after 30 min, a group of animals received a subcutaneous injection of 25 mg/kg (DHEA) repeated twice a day (3 days). Exploratory behavior and general activity were evaluated 24 h and 48 h after the treatments by the open field test. Then, brain cortex and cerebellum were used for immunoblotting analysis of PKCε level.

**Results:** DHEA administration showed a significant improvement of locomotor activity both 24 and 48 h after D-galactosamine treatment (^****^p < 0.0001) but did not ameliorate liver parenchymal degeneration. Western blot analysis revealed a reduced immunoreactivity of PKCε (^*^p < 0.05) following D-galactosamine treatment in rat cortex and cerebellum. After the addition of DHEA, PKCε increased in the cortex in comparison with the D-galactosamine-treated (^***^p < 0.001) and control group (^*^p < 0.05), but decreased in the cerebellum (^*^p < 0.05) with respect to the control group. PKCε decreased after treatment with NH_4_Cl alone and in combination with DHEA in both cerebellum and cortex (^****^p < 0.0001). MTS assay demonstrated the synergistic neurotoxic action of NH_4_Cl and glutamate pretreatment in cerebellum and cortex along with an increased cell survival after DHEA pretreatment, which was significant only in the cerebellum (^*^p < 0.05).

**Conclusion:** An association between the DHEA-mediated increase of PKCε expression and the improvement of comatose symptoms was observed. PKCε activation and expression in the brain could inhibit GABA-ergic tone counteracting HE symptoms. In addition, DHEA seemed to ameliorate the symptoms of HE and to increase the expression of PKCε in cortex and cerebellum.

## Introduction

Hepatic encephalopathy (HE) is a reversible neuropsychiatric syndrome characterized by an impaired brain function that generally occurs in patients suffering from chronic liver disease or acute liver failure (1, 2). Three different forms of HE have been identified: type A (associated with acute liver failure resulting from severe inflammatory and/or necrotic liver disease of rapid onset), Type B (resulting from portacaval shunt in absence of parenchymal liver disease) and Type C (accompanying chronic liver failure) (3).

Although the pathophysiological process of HE is poorly understood, the scenario of an impaired neurotransmission due to metabolic changes in liver and brain with a consequent systemic inflammatory response and blood–brain barrier alterations has been hypothesized (1). In addition, non-specific neurological and psychiatric manifestations ranging from attention, working memory, psychomotor speed, and visuospatial ability alteration to disorientation to time and space, inappropriate behavior, and acute confusional state with agitation or somnolence have also been documented in patients suffering from HE (4, 5). A poor survival and a high risk of recurrence strongly characterize HE if liver disease is not successfully treated (6). Nevertheless, also the mildest form is correlated to a reduced quality of life as well as to the increased risk to develop severe HE (1, 7). One of the main contributors to the alteration in neurotransmission and neurological function in HE is hyperammonemia (1, 8). In this sense, blood ammonia increase following impaired liver function can cause muscle wasting, neuronal electric activity, and astrocyte swelling (1, 9–12). Astrocyte swelling, due to an increased intracellular osmolarity caused by impaired ammonia metabolism and glutamine production, is supposed to be one of the possible explanations of brain edema that generally characterizes HE (11, 13).

Neurochemical, neurobehavioral, and electrophysiological studies revealed the involvement of GABA-ergic (8, 14), dopaminergic (15), and serotoninergic and glutamatergic (14) neurotransmitter systems in the neurotransmission and neurological impairment that characterize HE. It is well-known that neurosteroids play pivotal functions in CNS. In particular, dehydroepiandrosterone (DHEA) has different positive effects on the central nervous system (CNS) ranging from neuronal plasticity and survival to cognition and behavior (16). As far as type A and type C HE are concerned, it seems that the GABA_A_-ergic neurotransmission is activated by benzodiazepines (diazepam and *N*-desmethyldiazepam) and neuroactive steroids (allopregnanolone and pregnenolone) present in the brain, sera, and cerebrospinal fluid of patients (17, 18). Conversely, recent neurophysiological studies on HE revealed that reduced brain levels of DHEA, which is a negative allosteric modulator of GABA_A_ receptors, induced the stimulation of these latter with a consequent increase in GABAergic tone and neuroinhibition prevention (19).

Although these findings described a neuroprotective role of DHEA and partially explain its non-genomic molecular mechanism, its potential neuroprotection is still a matter of investigation in HE. Another potent modulator of GABA_A_ and glutamate (in particular NMDA) receptor activity involved in HE is the protein kinase C (PKC) (20, 21). In fact, hyperammonemia decreases PKC-mediated phosphorylation and alters the function of key proteins involved in the neurotransmission (Na+/K+-ATPase and NMDA receptors) (22) and glutamine transporter [system N (SN)1 and SN2] (23) contributing to the impairment of neuronal functions (21). In addition, the PKC epsilon (PKCε) is involved in the molecular events leading to the decrease in the number of GABA_A_ receptors through phosphorylation and activation of ATPase that regulates membrane fusion events (24). This finding is even more interesting if we consider that DHEA was able to interact with the PKC containing α, β, and γ isoforms and promote the translocation of PKC-β and -ζ from the cytosol to the membrane in rat adipocytes (25).

Given this information, in the present research project, we investigated the protective effect of DHEA in an animal model of HE, and the possible modulation of PKCε expression in different brain area.

## Materials and Methods

### Animals

Twenty-four, 7-week-old, male Sprague–Dawley rats (Charles River, Calco, Italy), with a body weight ranging from 200 and 220 g, were used. The animals were housed in an air-conditioned environment at 22 ± 1°C, with controlled humidity (60%), and a 12-h light/12-h dark cycle. Food and water were available *ad libitum*. Animal care and maintenance were conducted in accordance with the Italian law (D.L. n. 116/1992) and European legislation (EEC n. 86/609). The experimental designs and procedures received the approval of the Bioethical Committee of the Italian Institute of Health.

### Animal Protocol Design and Pharmacological Treatments

Eighteen rats were randomly divided into three groups of six animals each as given below:

Group I: control (saline only)

Group II: D-galactosamine

Group III: D-galactosamine + DHEA

D-galactosamine (Sigma-Aldrich, Milan, Italy) was solubilized in physiological solution and administered at a single dose of 2.5 g/kg by intraperitoneal injection. DHEA (Sigma-Aldrich, Milan, Italy) was solubilized in peanut oil and subcutaneously injected at the dose of 25 mg/kg twice a day.

It is well-known that the administration of D-galactosamine at the dose of 2.5 g/kg induced fulminant hepatic encephalopathy after 48 h from injection (14). For this reason, 48 h after the galactosamine treatment, all animals were sacrificed and the brains were isolated.

### Behavioral Investigation

The endpoints of functional neurotoxicity were investigated with the open field test. Particularly, the spontaneous motor activity expressed as the total distance traveled (cm), and the number of rears (when the rat reared upon its hind feet) were recorded at two different times:

(1) 24 h after the D-galactosamine treatment
(2) 48 h after the D-galactosamine treatment

For the test, a black painted arena of 100 × 100 cm square floor and 50 cm high walls was used. The test started by gently placing each animal in the center of the arena. Rats were continuously filmed for 10 min with a camera connected at the video tracking system (SMART 2.5 version, PanLab, Barcelona, Spain). Each rat was tested only once, and at the end of each trial, the open field was wiped clean with 10% ethanol solution to remove traces of the previous assay.

### Cell Cultures and Pharmacological Treatments

For primary rats, cerebellar granule neurons were prepared from 8-day-old Sprague–Dawley rat cerebella as previously described (26). Instead, primary cortical neurons were prepared from 0 to 2 days postnatal brain pups. For cerebellar granule neurons, the brains were extracted from the skull and the cerebellum was excised, minced, and treated as previously reported (26). Both primary granule cells and cortical neurons were dispersed with trypsin (0.25 mg/ml; Sigma, Italy) and plated at a density of 100–105 cells/cm^2^ on 35-mm petri dishes or 96 multi-well plates coated with poly-L-lysine (10 mg/ml; Merck Life Science S.r.l., Milano, Italy). Cells were cultured in basal Eagle's medium (BME) (Euroclone, Milano, Italy) supplemented with 10% bovine calf serum, 2 mM glutamine, and 100 mg/ml of gentamycin (Euroclone) and maintained at 37°C in 6% CO_2_.

After 24 h *in vitro*, in the cortical neurons, the medium was replaced with 1:1 mixture of BME and Neurobasal medium (Thermofisher, GIBCO, Monza, Italy) containing 2% B27 supplement, 1% antibiotic, and 0.25% glutamine [H_2_NCOCH_2_CH_2_CH(NH_2_)CO_2_H, molecular weight 146.14, Thermofisher Invitrogen, Monza, Italy]. At 5 days in culture (DIV 5), cytosine arabinofuranoside (Ara-C; C_9_H_13_N_3_O_5_, ≥ 90.0% purity, molecular weight 243.22, Merck Life Science S.r.l., Milano, Italy) was added at a final concentration of 1 mM, whereas in the cerebellar granule cells, Ara-C (1 mM) was added to all cultures 18–24 h after plating to inhibit glial proliferation. Cerebellar granule cells and cortical neurons were then pre-treated with DHEA (1 mM, C_19_H_28_O_2_, molecular weight 288.42) alone or in combination with PKCε inhibitor peptide (1 mM; C_37_H_65_N_9_O_13_, molecular weight 843.96, Santa Cruz, USA) and PMA (50 nM; C_36_H_56_O_8_, molecular weight 616.83, Merck Life Science S.r.l., Milano, Italy) on days 5 and 7, respectively, after plating. Based on previous dose/response (data not shown) on the same cell line, on days 6 and 8, the cells were treated with Glutamate (100 mM; C_10_H_16_MgN_2_O_8_ · 4H_2_O, ≥ 98.0% purity, molecular weight 388.61, Merck Life Science S.r.l., Milano, Italy) + ammonium chloride (10 mM; NH_4_Cl, ≥ 99.998% purity, molecular weight 53.49, Merck Life Science S.r.l., Milano, Italy) for further 24 h. The drugs were diluted in culture medium from stock solutions prepared in water or dimethyl sulfoxide [DMSO, (CH_3_)_2_SO, ≥99.9% purity, molecular weight 78.13, Sigma Aldrich]. DMSO at the same final dilution (0.2%) was used for control cells.

### Western Blot Analysis

Proteins from brain tissues, cerebellar granule cells, and cortical neurons of control and treated samples were obtained by lysing and homogenizing (in ice) the samples in RIPA buffer [50 mM Tris-HCl pH 7.4, 150 mM NaCl, 1% sodium deoxycolate, 1% Triton X-100, 2 mM phenylmethylsulfonyl fluoride (PMSF)] (C_7_H_7_FO_2_S, molecular weight 174.19, Merck Life Science S.r.l., Italy). The samples were then quantified using Bradford colorimetric method (Pierce, Rockford, USA) according to the manufacturer's protocol. Equal amount of protein, 0.4 μg/μl for each sample, was loaded onto a pre-cast 12% sodium dodecyl sulfate–polyacrylamide gel electrophoresis (SDS-PAGE) (Thermofisher Invitrogen, Monza, Italy) and electrophoretically transferred to nitrocellulose membrane (Thermofisher Invitrogen, Monza, Italy). Membrane was blocked in tris-buffered saline and Tween 20 (TBST) buffer (20 mM Tris- HCl, 0.5 M NaCl, and 0.05% Tween 20) containing 5% non-fat, dried, and incubated with primary antibody anti-PKCε (1 mg/ml, molecular weight 83 kDa, Upstate, USA) at 5°C overnight under gentle agitation. Membrane was then washed three times in TBST, incubated for 1.5 h with HRP-conjugated anti-mouse antibody (Cell Signaling, USA), and visualized using chemiluminescence method (Amersham, GE Healthcare Europe GmbH, Milan, Italy). The determination of relative protein expression was performed using densitometric analysis using a BioRad GS 690 Imaging densitometer with molecular analysis software (Life science, Milan, Italy). β-actin was used a loading control.

### Cell Viability Assay

After 8 days since the plating in the 96-well microplates, both cell cultures were pre-challenged with DHEA 1 μM and PMA (Merck Life Science S.r.l., Milano, Italy) 50 nM in aqueous solution. Cells were incubated for 24 h and then challenged with glutamate 100 μM (solubilized in HCl) and ammonium chloride 10 mM. The cell viability assay was carried out following another 24 h of incubation using the 3-(4,5-dimethylthiazolyl-2)-2,5-diphenyltetrazolium bromide (MTS) assay (Life Technologies, Monza, Italy). Viable cells were spectrophotometrically read at 490 nm.

### Histological Evaluation of Liver Tissues

The rat liver samples were washed with PBS, fixed with formalin, and serially sectioned into 5-μm slices after paraffin embedding. The slides were then dewaxed and stained using hematoxylin–eosin (HE) reagent. The histological changes of rat liver tissues were investigated under a light microscope.

### Statistical Analysis

Data were analyzed using GraphPad Prism8 software (GraphPad Software, Inc., La Jolla, CA, USA) and reported as the mean ± standard error of the mean. Differences in total distance traveled and rears were analyzed using a two-way analysis of variance (ANOVA) followed by Tukey's multiple comparisons test, with individual variances computed for each comparison.

Differences in PKCε expression in protein extract from control (*n* = 6), D-galactosamine-treated (*n* = 6), and D-galactosamine + DHEA-treated animals (*n* = 6) were performed in triplicate and analyzed by a one-way ANOVA followed by Tukey's multiple comparisons test, with a single pooled variance.

At the same time, differences between cortical neuronal cells and cerebellar granule neurons were performed in quadruplicate in three different experiments analyzed by a one-way ANOVA followed by Tukey's multiple comparisons test, with a single pooled variance.

As far as cell viability assays are concerned, each experimental condition is reported by the mean ± standard error of the mean of three different experiments done in quintuplicate and were analyzed by a one-way ANOVA followed by Tukey's multiple comparisons test, with a single pooled variance. ^*^p < 0.05 was considered significant.

## Results

### Behavioral and Histological Liver Tissue Evaluation

**Figure 1:**
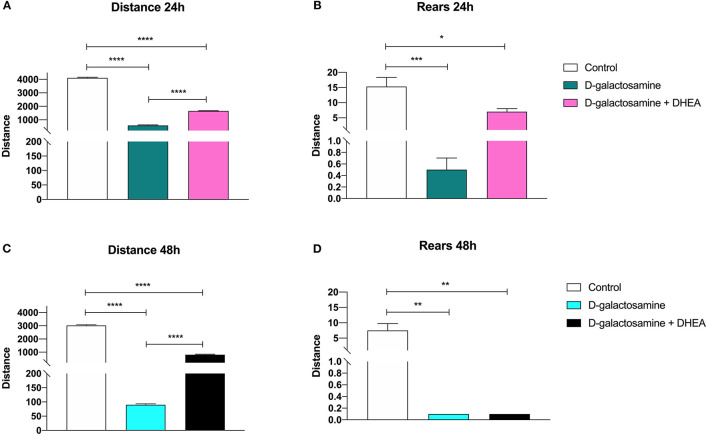
Effect of the administration of D-galactosamine alone and of D-galactosamine + DHEA on the total distance traveled and the number of rears in the open field test after **(A,B)** 24 h and **(C,D)** 48 h of treatment; *P < 0.05, **P < 0.01, ***P < 0.001, ****P < 0.0001.

In the evaluation of horizontal activity following the open field test, the groups of animals treated with D-galactosamine alone and treated with D-galactosamine + DHEA showed a significant reduction of the total distance traveled at both 24 and 48 h (^****^p < 0.0001) when compared with the control group (Figure 1).

However, the animals treated with D-galactosamine + DHEA showed a significant increase of the total distance traveled at both times 24 and 48 h (^****^p < 0.0001) in comparison with those treated with the D-galactosamine alone (Figure 1A).

The number of rears in the animals treated with D-galactosamine and D-galactosamine + DHEA were significantly lower in comparison with the control group at both 24 (^****^p < 0.0001 and ^**^p < 0.01, respectively) and 48 h (^**^p < 0.01) (Figure 1B).

**Figure 2:**
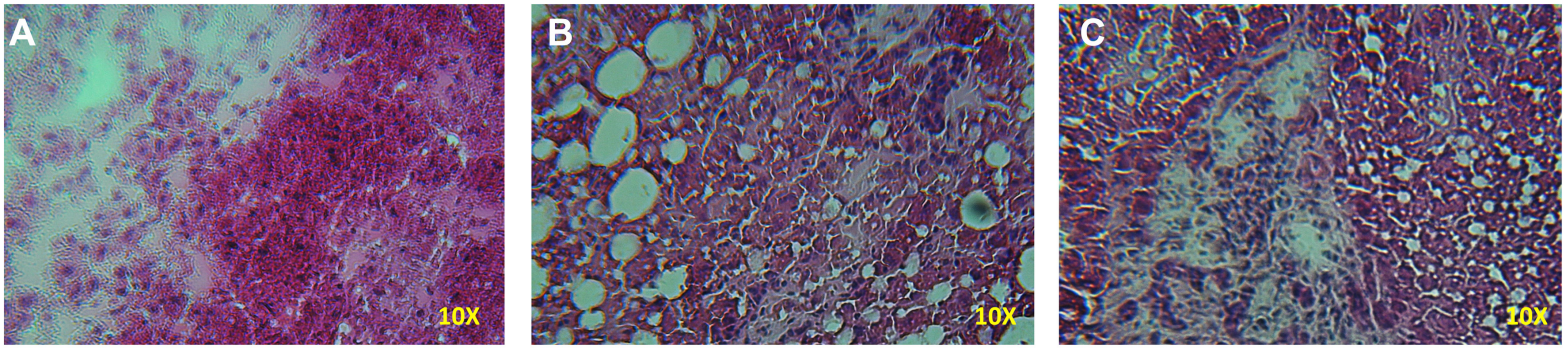
Histopathology of the liver samples stained with HE from rats treated with D-galactosamine and D-galactosamine + DHEA for 48 h. **(A)** Normal control group with proper architecture of the liver, **(B)** D-galactosamine-induced hepatotoxicity characterized by marked steatosis and hepatocyte degeneration, and **(C)** D-galactosamine + DHEA with steatosis and altered parenchymal structure.

A significant increase in the number of rears of the animals treated with D-galactosamine + DHEA with respect to the D-galactosamine-treated group (^*^p < 0.05) was observed at 24 h (Figure 1B). After 48 h of treatment, DHEA was unable to increase the number of rears in comparison to the D-galactosamine alone; this could be due to the degenerative effect promoted by D-galactosamine on liver parenchyma, where DHEA did not exert any significant protective effect (Figures 2B,C).

Indeed, as shown in Figure 2, D-galactosamine was able to alter the architecture of the liver by inducing hepatocyte degeneration and a dramatic increase of steatosis. The treatment with DHEA did not ameliorate the parenchymal degeneration, and the samples present steatosis and an altered cellular architecture.

### Evaluation of the PKCε Expression in the Cortex and Cerebellum Tissue and Cells

**Figure 3:**
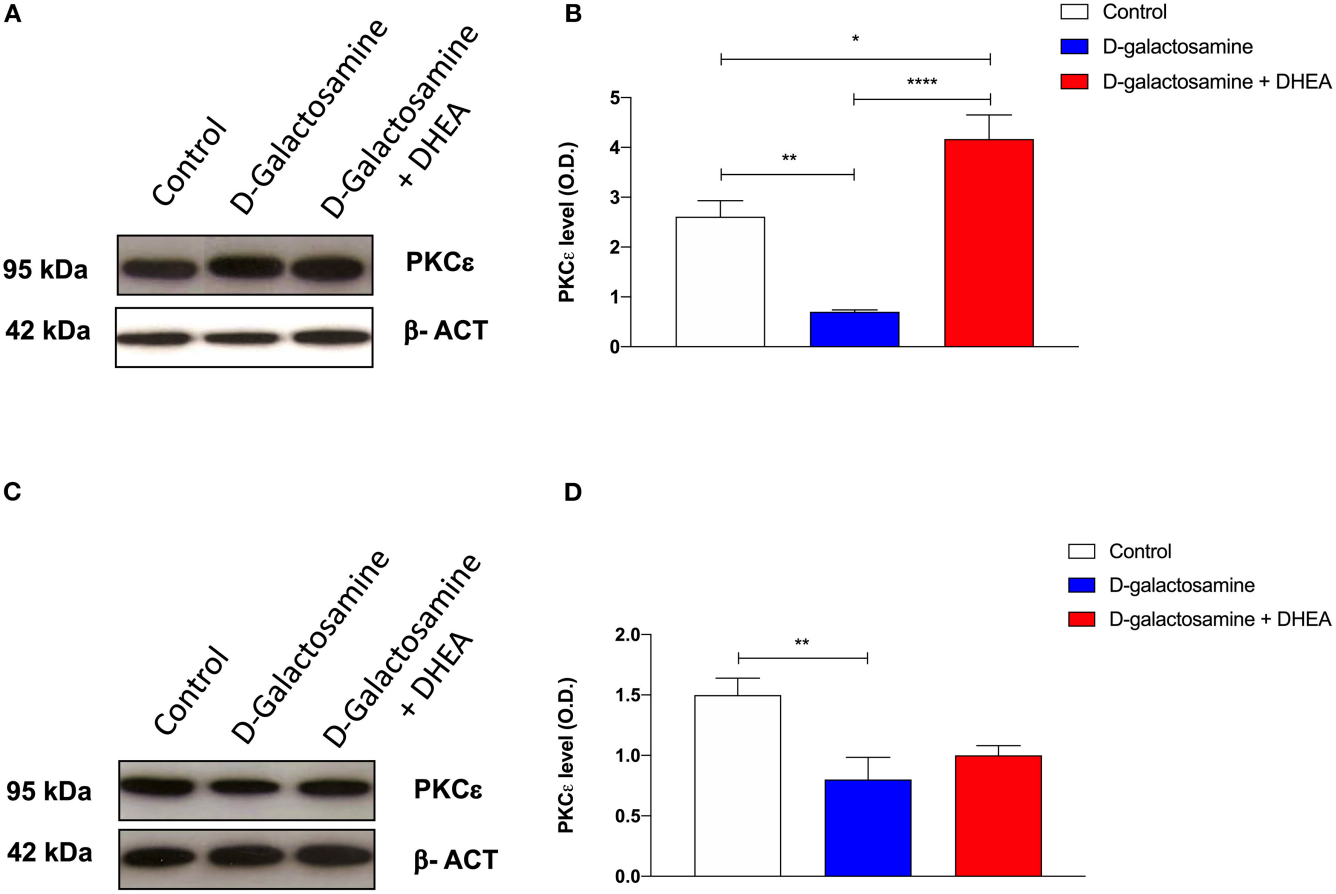
Western blot analysis of cortex **(A)** and cerebellum **(C)** PKCε expression after 48 h of treatment with saline (control), D-galactosamine, and D-galactosamine + DHEA (β-actin was used as internal standard) and their respective representative blots **(B,D)**; *P < 0.05, **P < 0.01, ****P < 0.0001. Western blot pictures were sliced since cortex and cerebellum samples were loaded in wells far from the control.

Immunoblot analysis of PKCε protein expression in rat cortex and cerebellum tissue samples, revealed the presence, in both membrane preparations, of the 95-kDa protein, corresponding to PKCε (Figures 3A,C). In both cortex and cerebellum, D-galactosamine-treated rats significantly reduced (^*^p < 0.05) the immunoreactivity of PKCε compared to that of the control (Figures 3B,D).

In particular, band spectrophotometric analysis of the protein showed a one-fold expression reduction in cortex and a 0.75-fold in cerebellum. As far as cortex tissue samples are concerned, after the addition of the DHEA, the protein level of PKCε significantly increased in comparison with both the D-galactosamine (^***^p < 0.001) and control group (^*^p < 0.05) (Figure 3B). Indeed, D-galactosamine + DHEA treatment expression value showed a two-fold increase with respect to the control and a three-fold increase with respect to the D-galactosamine group, thus demonstrating that DHEA was able to stimulate PKCε expression in brain cortex area.

On the contrary, densitometric analysis performed on cerebellum protein tissue extracts showed that DHEA treatment failed to restore the protein concentration with respect to that of the control group, although a 0.25-fold increase with respect to the D-galactosamine group was observed.

### DHEA and Phorbol 12-Myristate 13-Acetate (PMA) Effects on Glutamate- and Ammonium Chloride-Induced Toxicity in Cortical Neuronal Cells and Cerebellar Granule Neurons

The results obtained on the HE-animal model prompted us to investigate the role of PKCε in neuroprotection of cortical and cerebellar granular neurons challenged with a combined treatment with glutamate and ammonium chloride. The reason for the choice of ammonia and glutamate plus ammonia treatments was based on a previous report, where researchers observed a dramatic increase in ammonia in CNS in D-galactosamine-induced HE in rats (27), along with an increase in glutamate in the intersynpatic cleft (8, 11).

**Figure 4:**
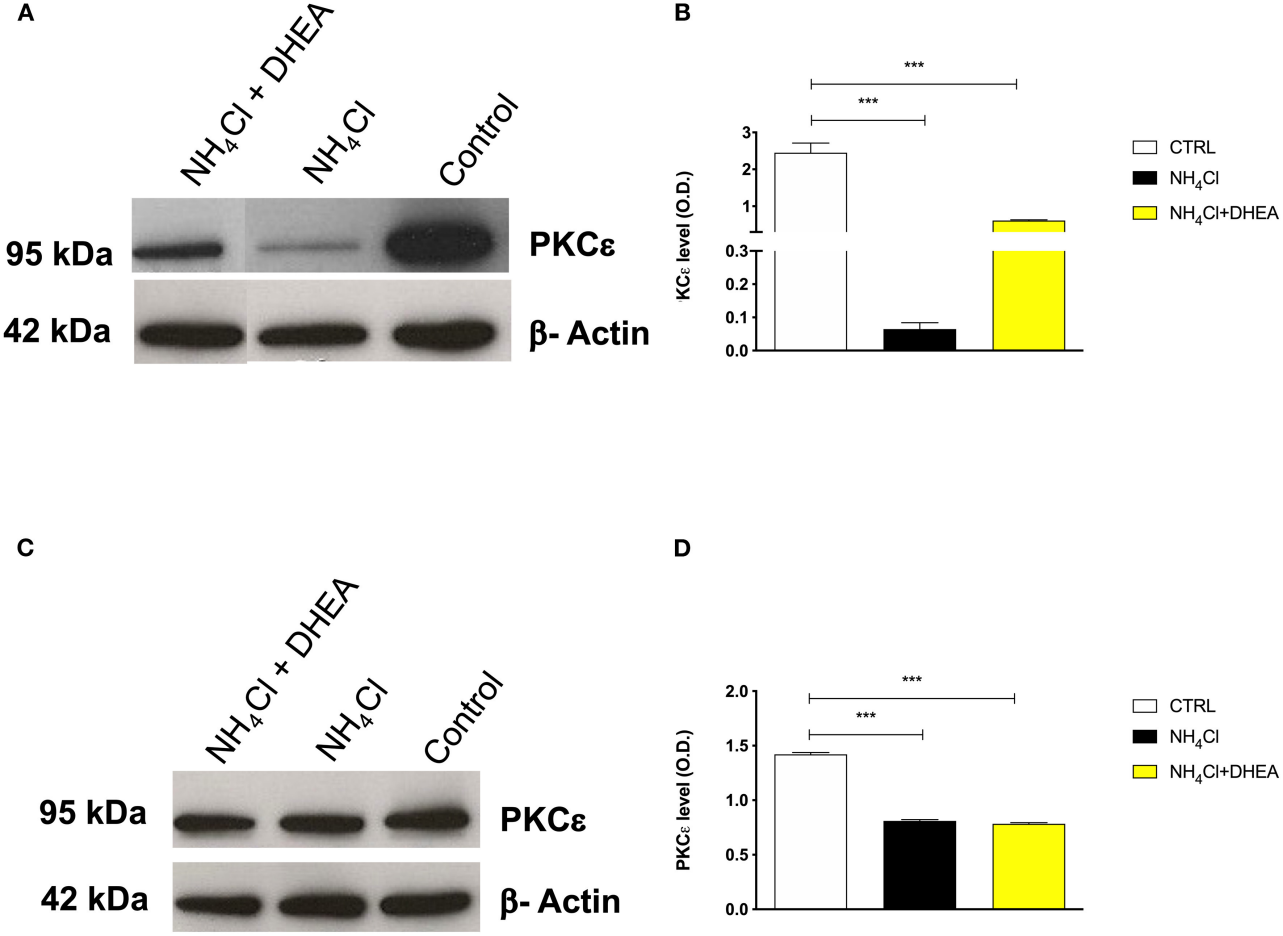
Western blot analysis of cortex **(A)** and cerebellum **(C)** PKCε expression after 48 h of treatment with saline (control), NH_4_Cl, and NH_4_Cl + DHEA (β-actin was used as internal standard) and their respective representative blots **(B,D)**; ***p < 0.001. Western blot pictures were spliced since cortex and cerebellum samples were loaded in wells far from the control.

As expected, the signal of PKCε decreased significantly after treatment with NH_4_Cl alone (0.75- and 10-fold) and in combination with DHEA (0.75- and 1.5-fold) (1.42 ± 0.01 vs. 0.81 ± 0.01 and 0.78 ± 0.01, respectively; ^****^p < 0.0001) in both cerebellum (Figures 4C,D) and cortex tissue extracts (2.45 ± 0.26 vs. 0.06 ± 0.02 and 0.61 ± 0.02, respectively; ^****^p < 0.0001) (Figures 4A,B). As observed in the animal model, the effect of NH_4_Cl in the cerebellar cortical cells produced a decrease in the PKCε expression, which was not reverted by DHEA. In the cortical neurons instead, the NH_4_Cl + DHEA challenge improved the PKCε expression with respect to the NH_4_Cl alone. We hypothesized that this could be due to an increase in transcription elicited by DHEA.

**Figure 5:**
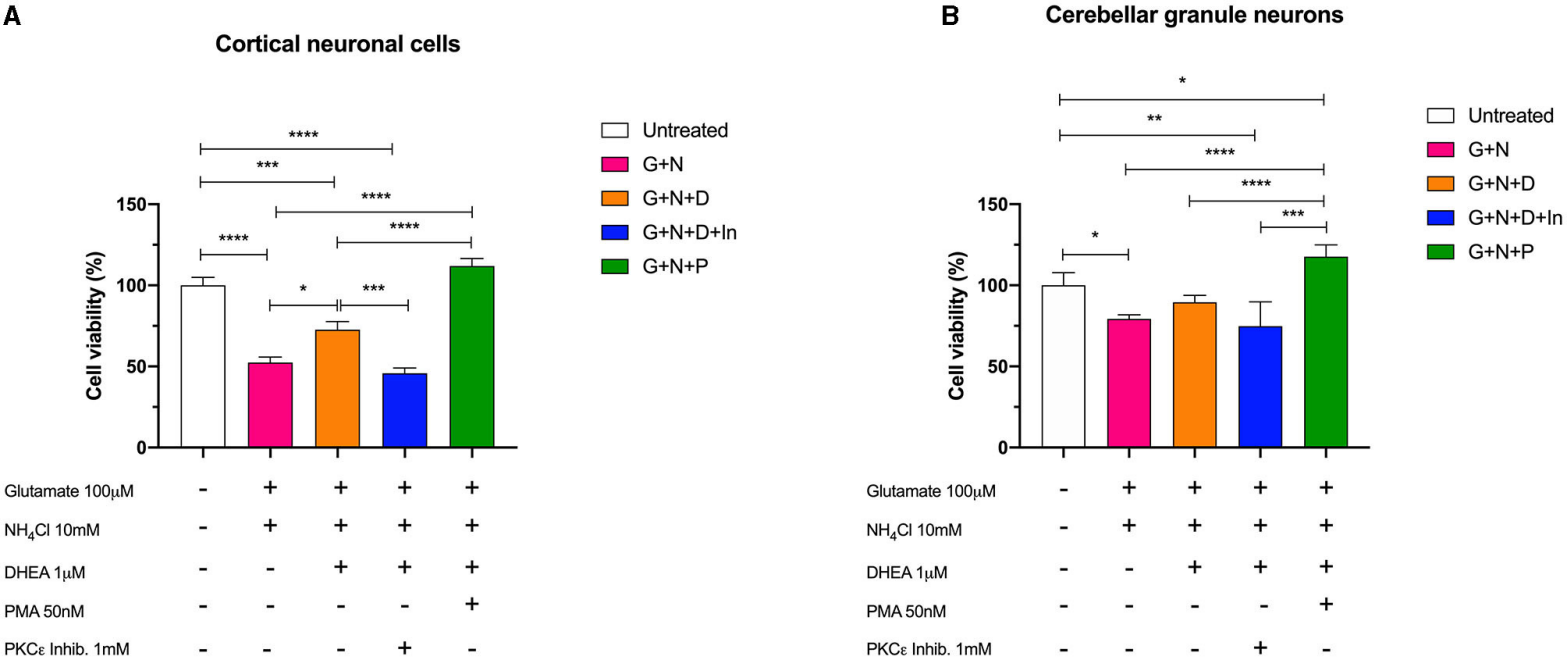
Cell viability assay of **(A)** cerebellar cells and **(B)** cortical cells following 24 h of incubation with glutamate (G), ammonium chloride (N), DHEA (D), PMA (P), and PKCε inhibitor (In); ^*^p < 0.05, ^**^p < 0.01, ^***^p < 0.001, ^****^p < 0.0001.

We then treated the cells with the combination of glutamate (100 mM) and ammonium chloride (10 mM) for 24 h, with or without DHEA (1 mM) and PMA (50 nM) pretreatment and with a PKCε-specific inhibitor peptide (1 mM) to better characterize and describe further possible synergistic combinations. The results obtained by MTS assay in both cerebellum and cortex demonstrated the synergistic neurotoxic action of ammonium chloride and glutamate (N + G). In fact, the cells treated with this combination showed a significantly reduced cell viability characterized by a decreased absorbance compared to the control cells (untreated). Conversely, pretreatment of cells with glutamate, ammonium chloride, and DHEA (G + N + D) significantly increased (^*^p < 0.05) the cell survival with respect to N + G, thus demonstrating the protective effect of DHEA (Figure 5).

Furthermore, a significant decrease (^***^p < 0.001) in the cell viability was observed in cells treated with ammonium chloride, glutamate, and PKCε inhibitor (G + N + D + In), possibly indicating a synergistic activity due to the neurotoxic effect of glutamate and ammonia, and the reduction of metabolic activity of PKCε. Interestingly, cell viability of cortical neuronal cells pretreated with glutamate, ammonium chloride, and PMA (G + N + P) was significantly increased compared to the other treatments (^****^p < 0.0001) and slightly, but not significantly, increased with respect to the control (untreated). In cerebellar granule neurons, a similar trend for the (G + N + P) treatment with respect to the other treatments was observed; however, a significant increase (^*^p < 0.05) with respect to the control (untreated) was further observed. This result is compatible with the intrinsic activity of PMA, which is an activator of PKC, including the γ isoform, and acts by stimulating the metabolic activity of cells. Moreover, the co-treatment with DHEA and PKCε inhibitor brings the value of cell viability back to that of the cells treated with glutamate and ammonia, in both cortical and granule cells. These latter data confirmed the neuroprotective role played by DHEA through the involvement of the modulation of PKCε.

## Discussion

As previously described, HE is a severe neuropsychiatric syndrome that arises during the presence of severe liver damage and is characterized by a general depression of the CNS induced by an increase of the GABA-ergic tone. In particular, this hypertonia of the GABA-ergic system can be interpreted as the final result of a series of enzymatic, metabolic, and degenerative modifications that occur in astrocytes and in neurons as well.

Although the pathogenesis of HE is not yet fully known, the accumulation of ammonium in the brain has been identified as the main factor able to trigger biochemical alterations that lead to CNS depression. The increase in GABA-ergic tone along with the degeneration of astrocytic cells contributes to the onset of HE. The neuronal alterations seem to be identified in an imbalance between inhibitory and excitatory systems.

It is widely demonstrated that the neurosteroid DHEA performs a neuro-protective action in the various models of insult (28). This action seems to take place with multiple action mechanisms both at ion channels and cell metabolism level. However, despite this evidence, it has not been elucidated yet whether there is a single pathway modulated by DHEA depending on the experimental insult.

The so-called novel ε isoform of PKC is particularly present within the CNS, more specifically at the level of the nerve endings of the cerebellum, hippocampus, and cortex where it is mostly expressed (29, 30). In particular, in these brain areas, the PKCε, through its catalytic activity, negatively modulates the activity of the GABA_A_ receptor complex through a decrease in the Cl^−^ currents induced by GABA. Since it has been reported that DHEA was able to activate typical and atypical PKC isoforms such α, β, and ζ in different tissues (31, 32), we sought a potential modulation of PKCε exerted by DHEA in our HE model, therefore unveiling a possible new molecular mechanism of DHEA neuroprotection.

Our results revealed that DHEA action significantly improved locomotor activity after 24 h with respect to the animals treated with D-galactosamine-induced hepatic encephalopathy.

Indeed, in animals treated with the DHEA, the ability of the animals to increase the total distance traveled was increased significantly. This result is in agreement with a decrease of rat's stress/anxiety level, since it is well-known that benzodiazepine, such as diazepam and 5-HT_1A_ agonist, was able to increase the locomotion in the open field experimental model (33). It is noteworthy that this parameter is of importance in HE since it has been demonstrated that HE mimics all the major psychiatric syndromes (34).

This result, together with the finding that DHEA protects the cultured neurons from the toxicity elicited by NH_4_Cl, suggests that the effect of the neurosteroid could be ascribed to both its neuroprotective effect and to an increase in PKCε expression.

Indeed, the PKCε protein expression increased in both cerebellar and cortical tissue in DHEA-treated animals compared to D-galactosamine although to different extents. The effect was particularly relevant in cerebral cortex tissue extracts, where the DHEA stimulated the expression of PKCε exceeding the level of the non-treated animals. These findings strongly suggest a direct modulation elicited by the neurosteroid on the PKCε activity and/or expression.

In fact, although the *in vitro* results seem to confirm the data obtained in the HE animal model, regarding the reduction of the PKCε expression in both cortical and cerebellar granule neuron cells following the glutamate + ammonium chloride treatment, the treatment with DHEA did not reflect precisely the results obtained in the animal model. Indeed, whereas we found an increase in the protein expression following the DHEA treatment in cortical neuronal cells, in the cerebellar granule neurons, this increment was not detectable. This discrepancy could be addressed to the intrinsic difference between the two models, although a post-translational regulation, rather than an PKCε gene expression modulation, cannot be excluded.

Although this finding needs further investigations, the results obtained on cell viability indicate a neuroprotection elicited by DHEA, through the involvement of PKCε.

Indeed, pretreatment of cerebellar neurons and granule cells with DHEA determined an increase of survival in comparison to the effect exerted by ammonium chloride and glutamate neurotoxins. Moreover, the use of a PKC activator, PMA, also led to a cell survival increase supporting the idea of a direct involvement of PKC activation. Interestingly, the treatment with combination of DHEA and PKCε inhibitor led to a decreased cell survival, reinforcing the hypothesis that the neuroprotective action of DHEA could occur through the involvement of the PKCε. In this context, it is reasonable to hypothesize that DHEA might decrease the GABA-ergic hypertonia by increasing the PKCε.

In fact, it has been shown that in knockout experiments for the gene encoding PKCε, there was an increase in the sensitivity of the GABA_A_ receptor to its allosteric modulators such as benzodiazepines, barbiturates, and neurosteroids, in brain areas including cerebellum and cortex (35).

Although more experiments are necessary to better understand the molecular mechanism, which involves DHEA in the PKCe translocation, our results showed, for the first time, a neuroprotective effect of DHEA through the stimulation of the PKCε, suggesting that molecules able to promote the expression of such protein could be useful for the symptomatic treatment of HE.

## Conclusions

In summary, the results obtained show an association between the DHEA-mediated increase in PKCε expression and the improvement of comatose symptoms of the HE animal model. This finding supports the idea that PKCε activation and expression at the brain level significantly inhibit GABA-ergic tone counteracting HE symptoms. In addition, our results have clearly shown that DHEA was able to ameliorate the symptoms of HE in animal models and also to increase the expression of PKCε in rat brain cortex and cultured primary neuronal cells as well.

## Data Availability Statement

The raw data supporting the conclusions of this article will be made available by the authors, without undue reservation.

## Ethics Statement

The animal study was reviewed and approved by Bioethical Committee of the Italian Institute of Health.

## Author Contributions

LC contributed to the conception and design of the study. LC, MZ, RA, LR, and GC performed the experiments. AD and CM performed the statistical analyses. AD and LC wrote the first draft of the manuscript. All authors contributed to manuscript revision, read, and approved the submitted version.

## Conflict of Interest

The authors declare that the research was conducted in the absence of any commercial or financial relationships that could be construed as a potential conflict of interest.

## Publisher's Note

All claims expressed in this article are solely those of the authors and do not necessarily represent those of their affiliated organizations, or those of the publisher, the editors and the reviewers. Any product that may be evaluated in this article, or claim that may be made by its manufacturer, is not guaranteed or endorsed by the publisher.
