# Supplementary figures and images for: Possible Association Between DHEA and PKCε in Hepatic Encephalopathy Amelioration: A Pilot Study
Source: Front Vet Sci. 2021 Sep 28;8:695375. doi: 10.3389/fvets.2021.695375 (PMC8505975; doi:10.3389/fvets.2021.695375)

Supplementary Material

## Supplementary Figures


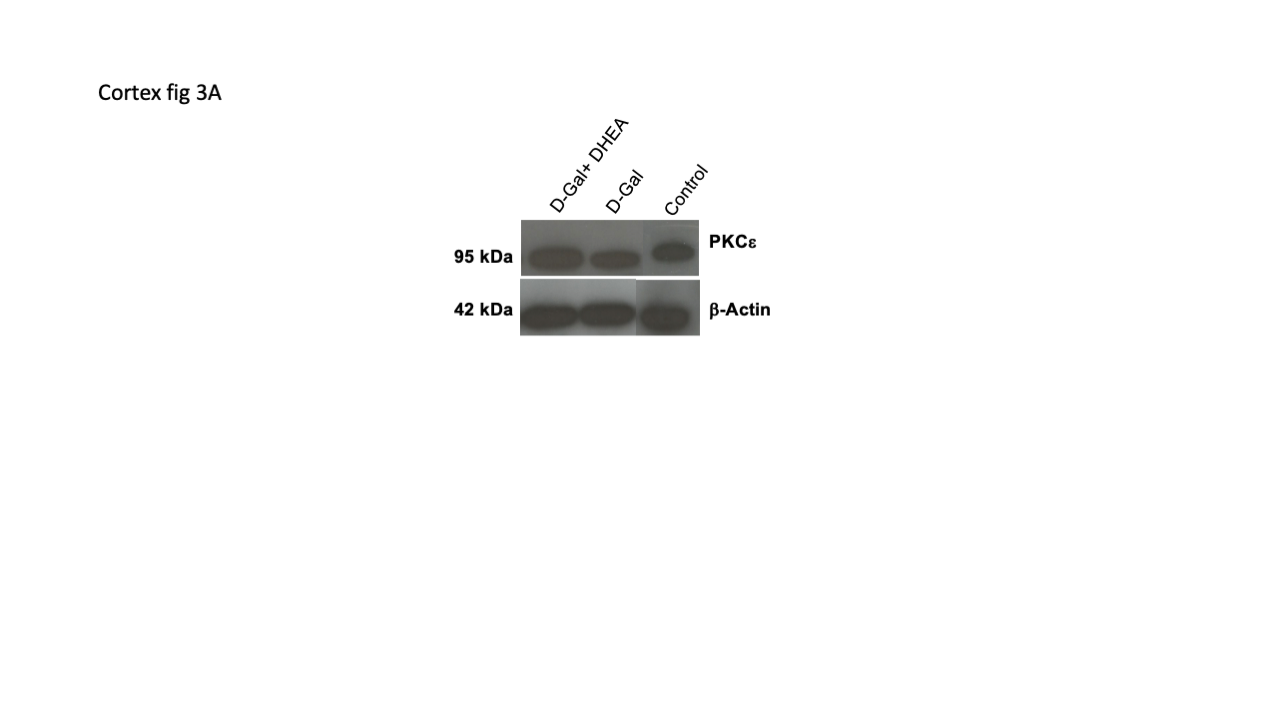

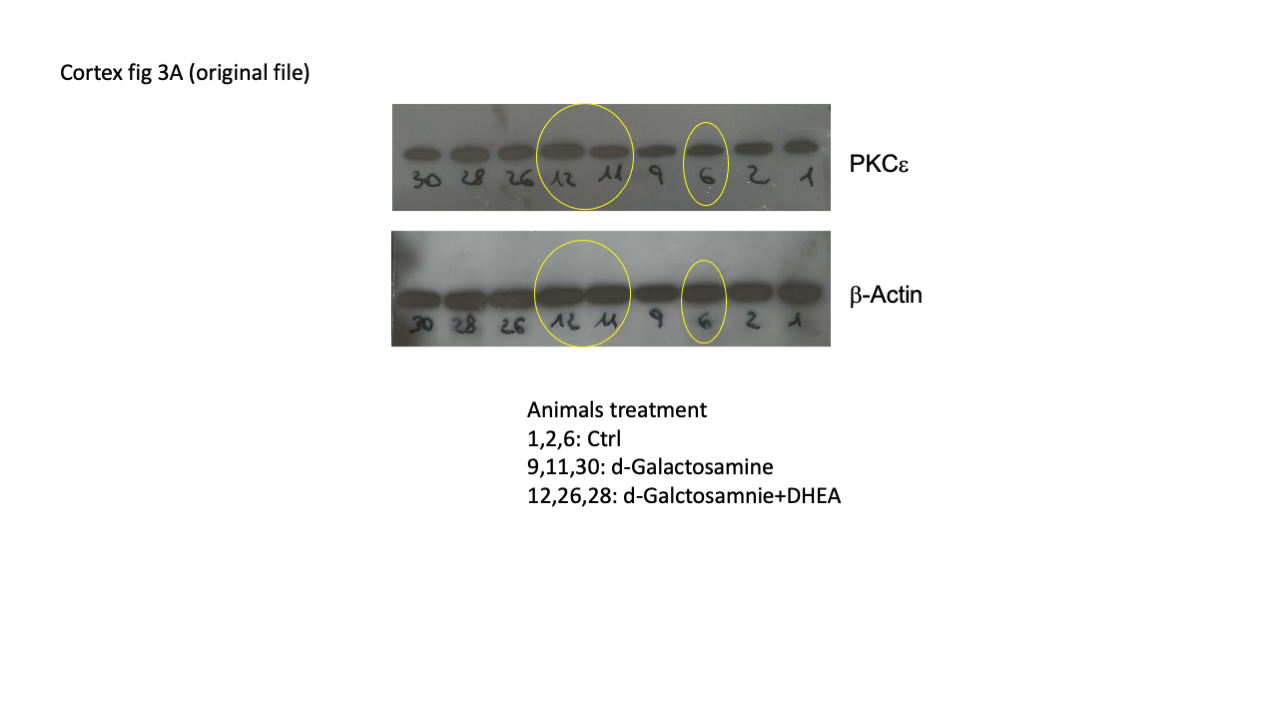


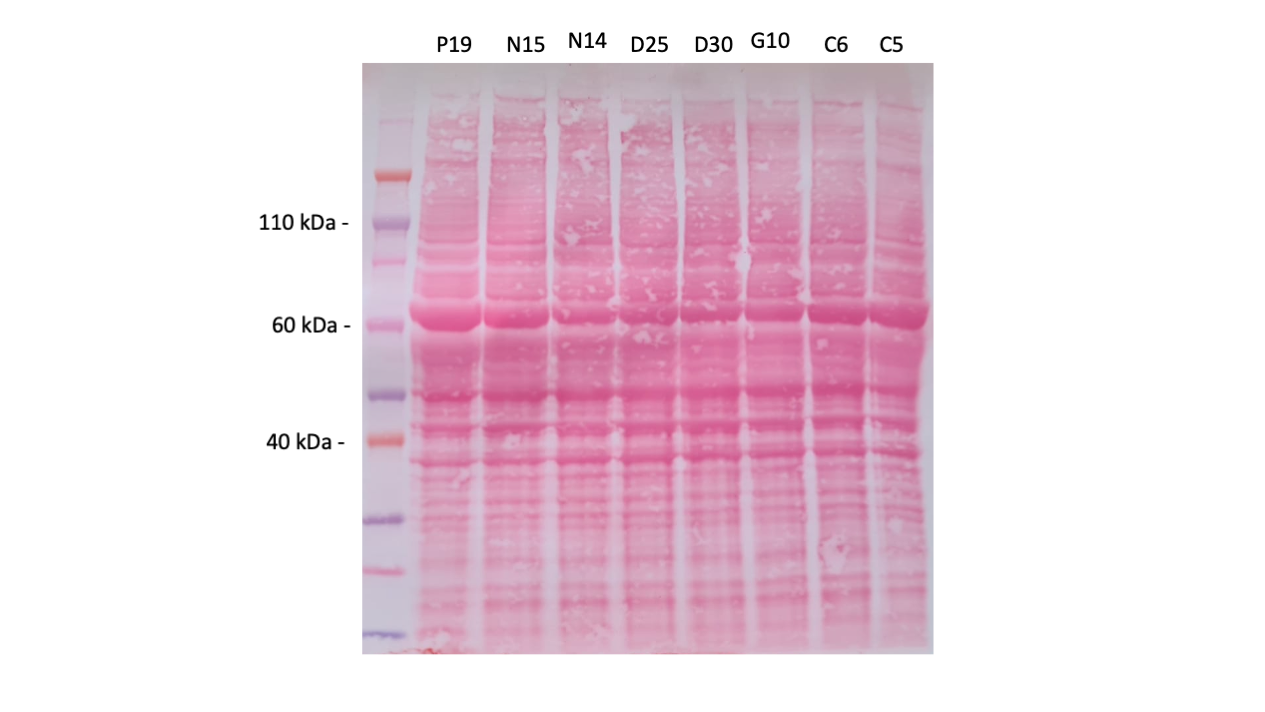


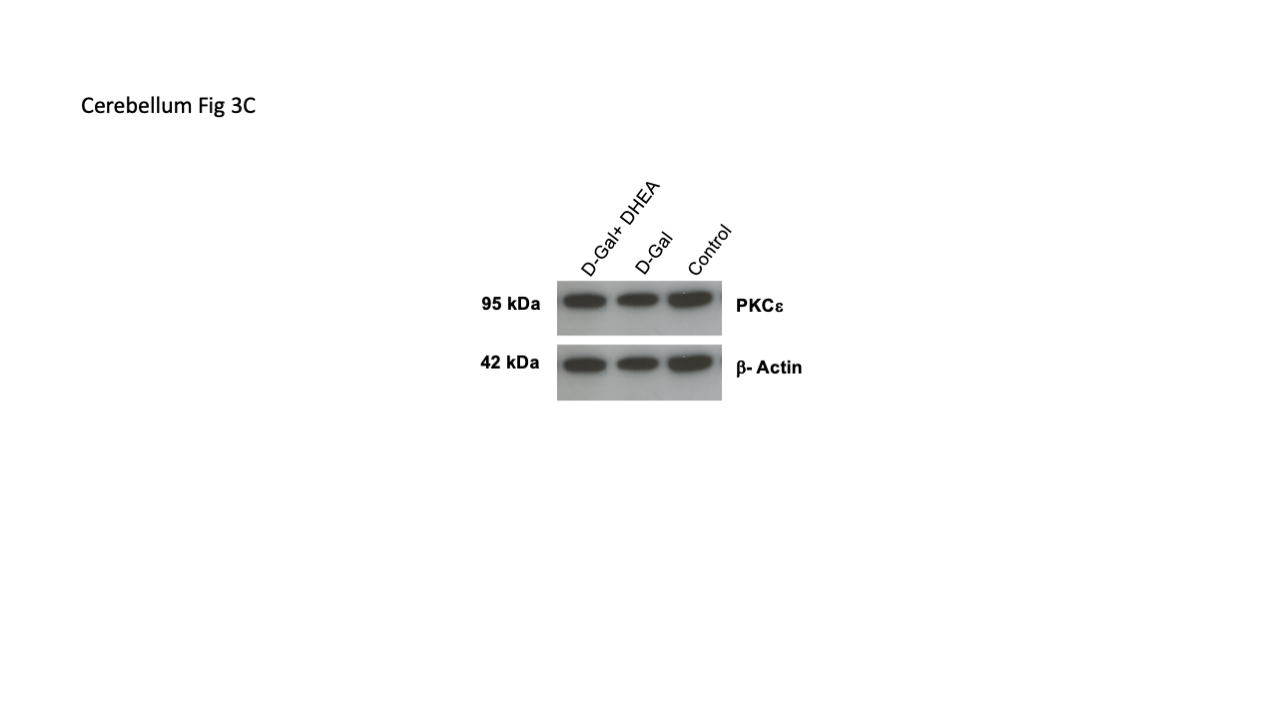

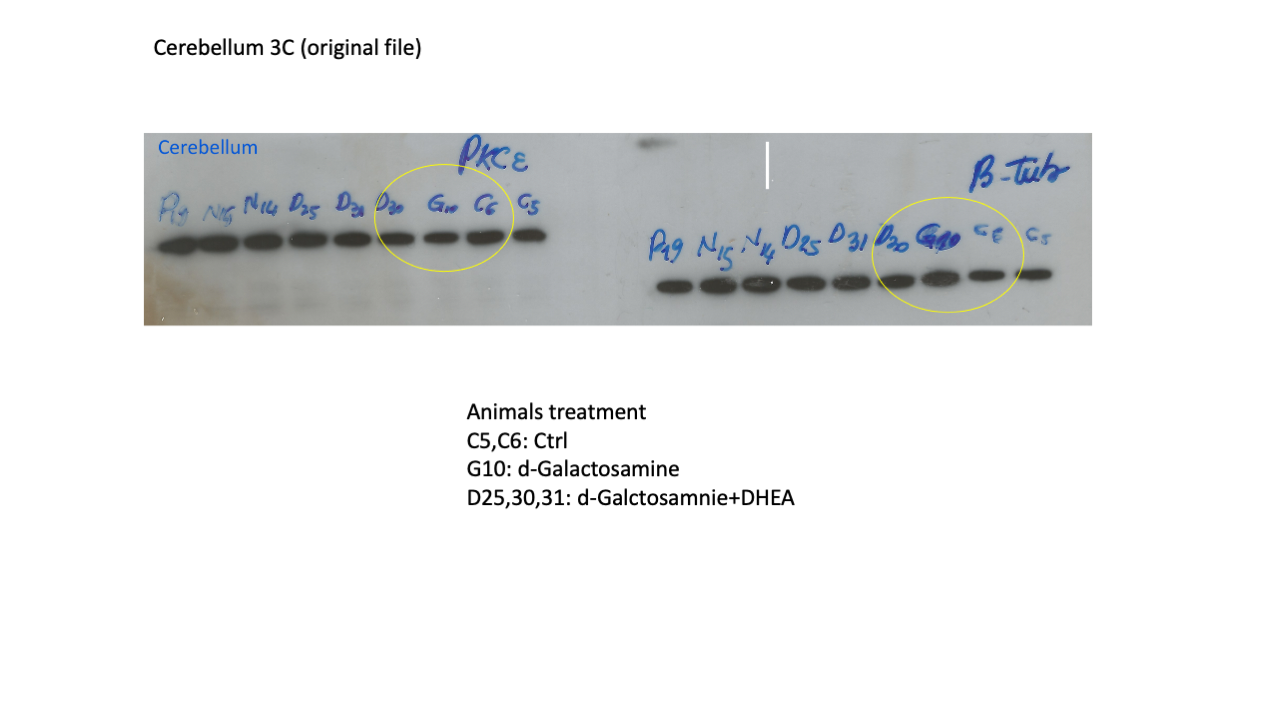


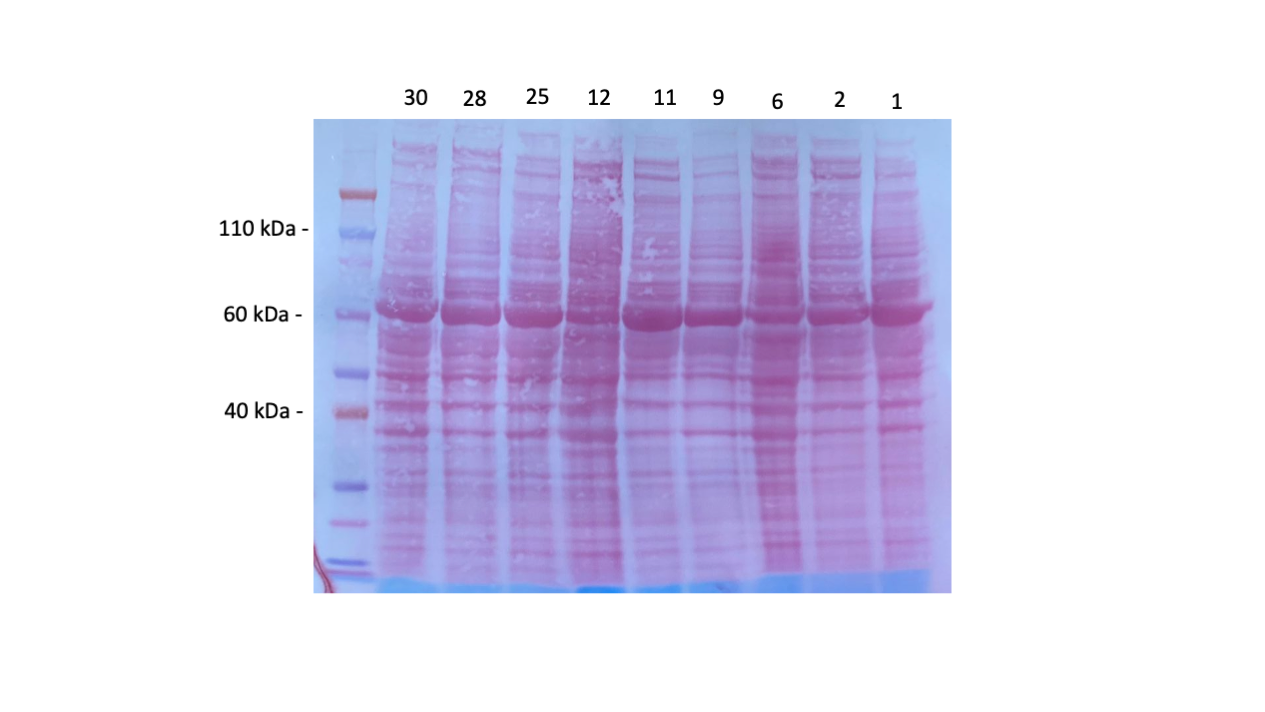


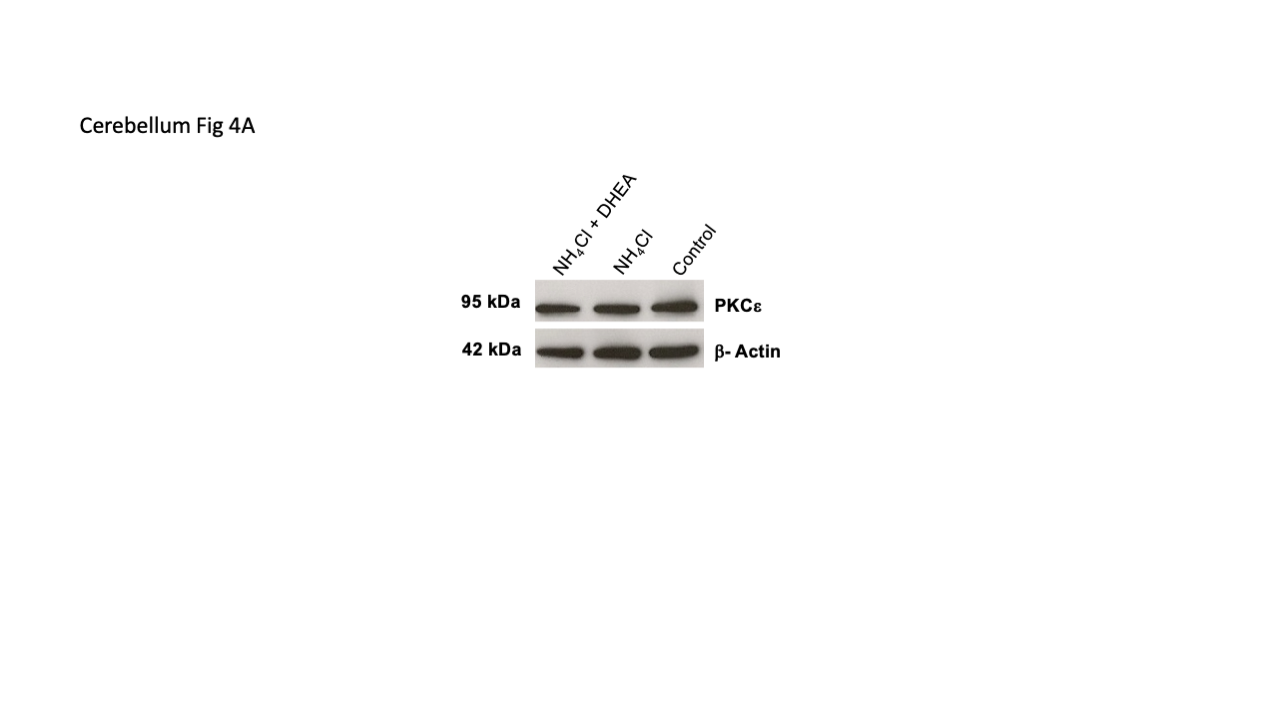

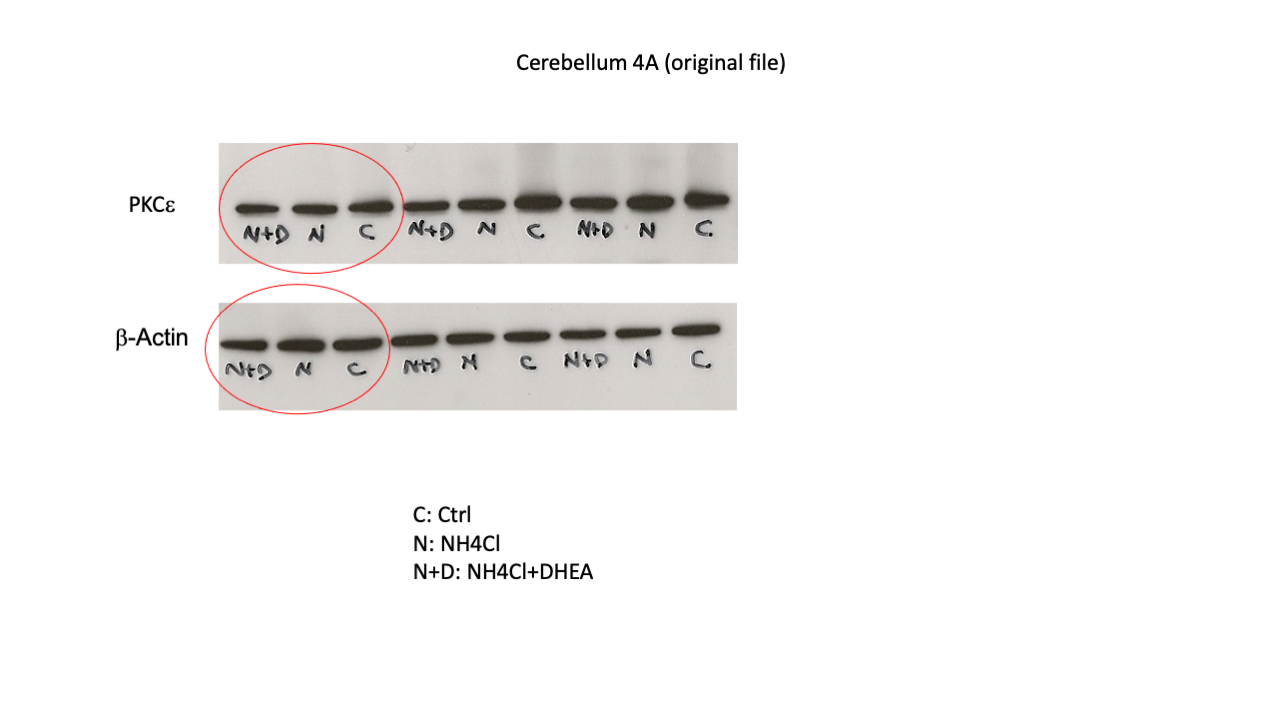


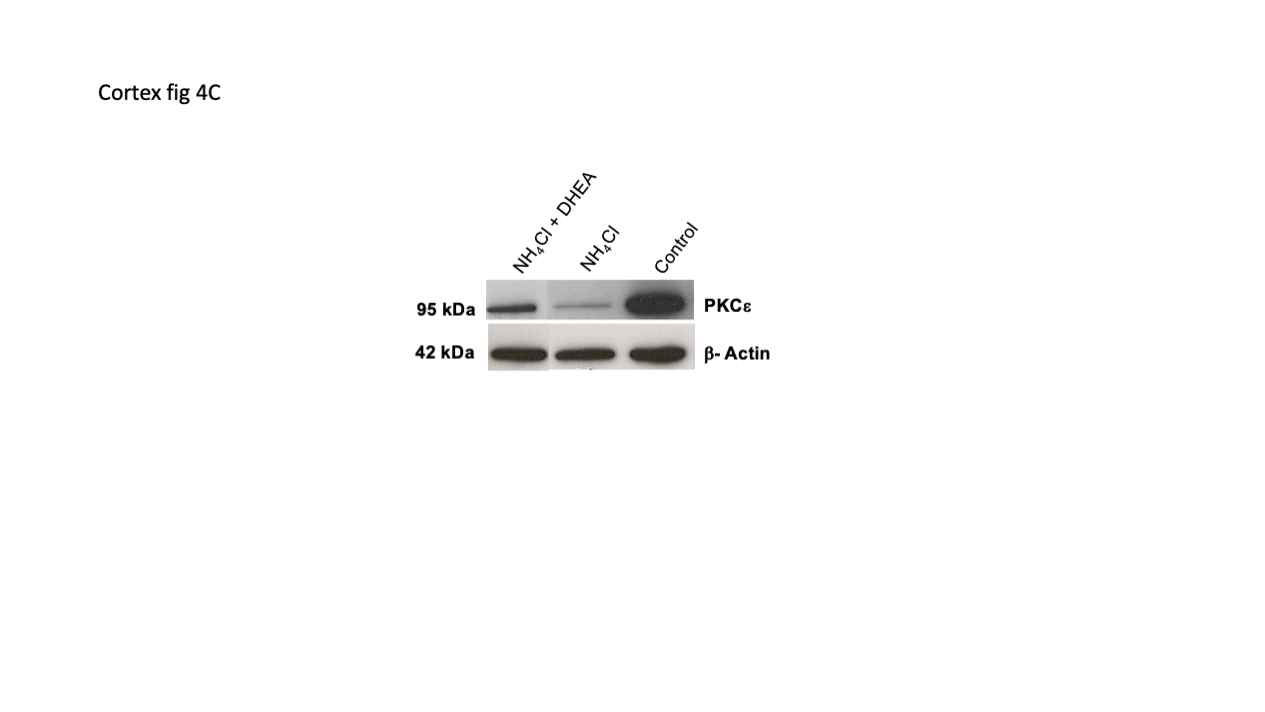

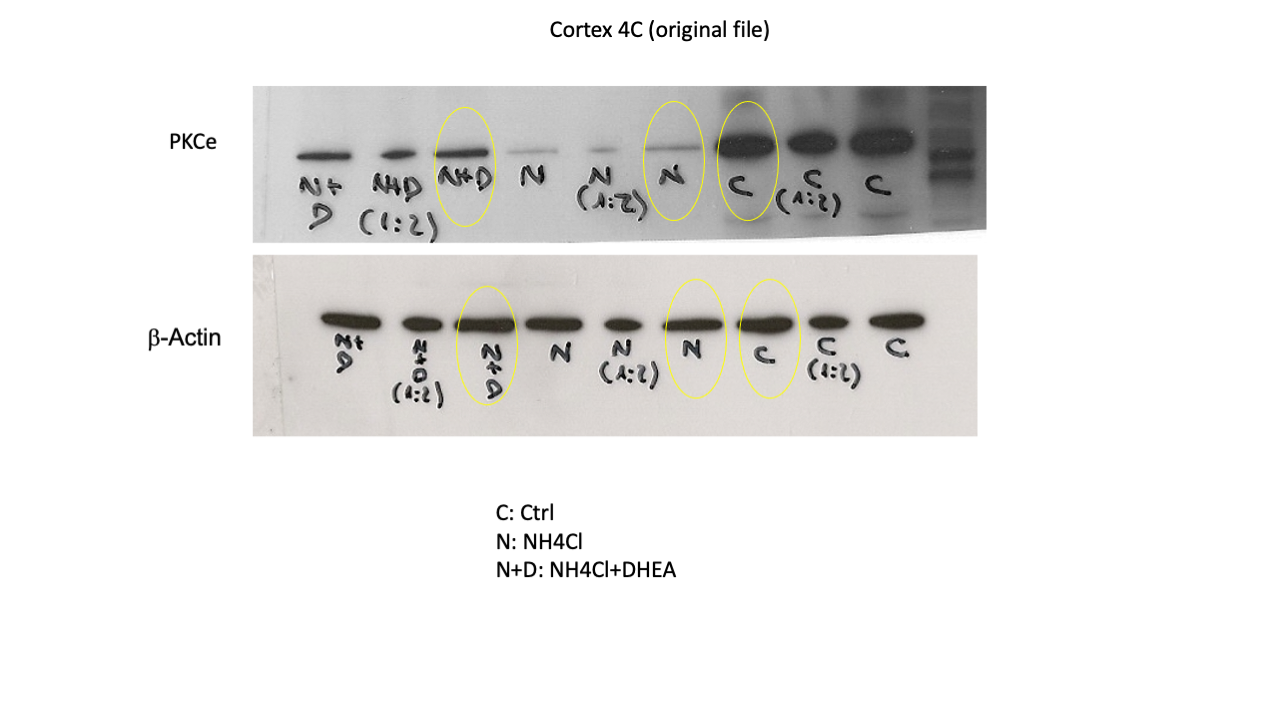


Fig 5


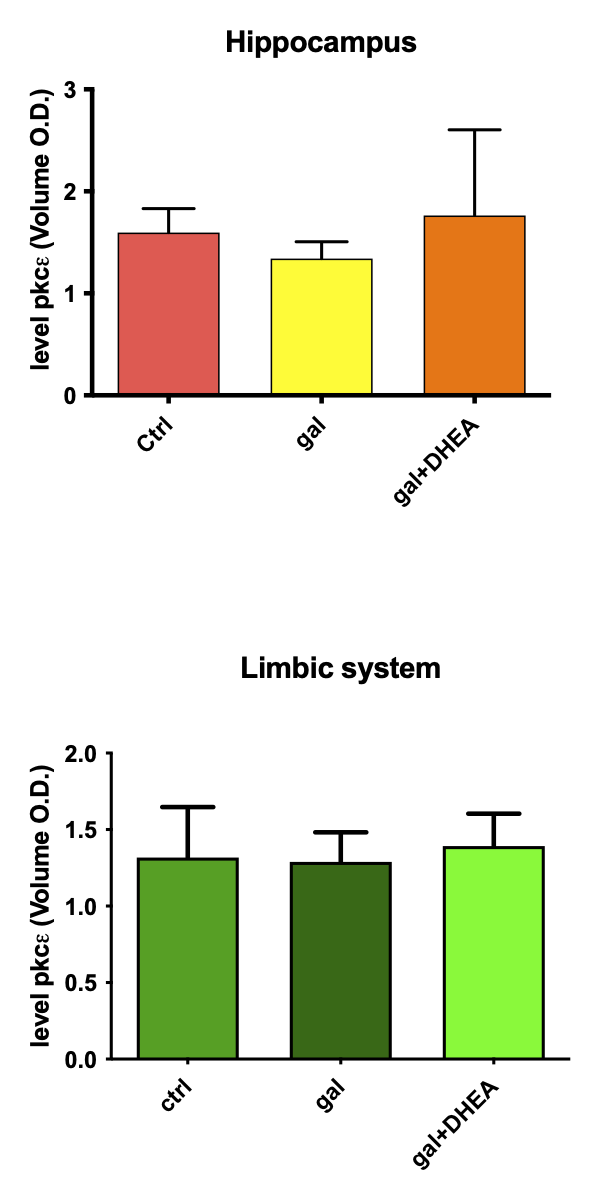

Supplement: Supplementary file 1 [file Data_Sheet_1.docx]
